# Shared and Unique Patterns of Embryo Development in Extremophile Poeciliids

**DOI:** 10.1371/journal.pone.0027377

**Published:** 2011-11-07

**Authors:** Rüdiger Riesch, Ingo Schlupp, R. Brian Langerhans, Martin Plath

**Affiliations:** 1 Department of Biology & W. M. Keck Center for Behavioral Biology, North Carolina State University, Raleigh, North Carolina, United States of America; 2 Department of Zoology, University of Oklahoma, Norman, Oklahoma, United States of America; 3 Department of Ecology & Evolution, Goethe–University Frankfurt, Frankfurt, Germany; Ecole Normale Supérieure de Lyon, France

## Abstract

**Background:**

Closely related lineages of livebearing fishes have independently adapted to two extreme environmental factors: toxic hydrogen sulphide (H_2_S) and perpetual darkness. Previous work has demonstrated in adult specimens that fish from these extreme habitats convergently evolved drastically increased head and offspring size, while cave fish are further characterized by reduced pigmentation and eye size. Here, we traced the development of these (and other) divergent traits in embryos of *Poecilia mexicana* from benign surface habitats (“surface mollies”) and a sulphidic cave (“cave mollies”), as well as in embryos of the sister taxon, *Poecilia sulphuraria* from a sulphidic surface spring (“sulphur mollies”). We asked at which points during development changes in the timing of the involved processes (i.e., heterochrony) would be detectible.

**Methods and Results:**

Data were extracted from digital photographs taken of representative embryos for each stage of development and each type of molly. Embryo mass decreased in convergent fashion, but we found patterns of embryonic fat content and ovum/embryo diameter to be divergent among all three types of mollies. The intensity of yellow colouration of the yolk (a proxy for carotenoid content) was significantly lower in cave mollies throughout development. Moreover, while relative head size decreased through development in surface mollies, it increased in both types of extremophile mollies, and eye growth was arrested in mid-stage embryos of cave mollies but not in surface or sulphur mollies.

**Conclusion:**

Our results clearly demonstrate that even among sister taxa convergence in phenotypic traits is not always achieved by the same processes during embryo development. Furthermore, teleost development is crucially dependent on sufficient carotenoid stores in the yolk, and so we discuss how the apparent ability of cave mollies to overcome this carotenoid-dependency may represent another potential mechanism explaining the lack of gene flow between surface and cave mollies.

## Introduction

### 1.1. Ecological speciation in extremophile poeciliids

During ecological speciation reproductive isolation evolves as a consequence of ecologically based divergent selection [1]–[3]. Most studies on ecological speciation in animals have focused on divergent selection as a result of biotic interactions, with populations differing, for example, in resource use [4]–[7], predation pressures [8], [9], or parasite exposure [10]–[12]. On the other hand, the concept of stress and maintenance of homeostasis through adaptation to abiotic habitat parameters is often ignored in the study of speciation in animals and more emphasis is put on its role in population decline and extinction [13], even though stressful environments have long been known to be associated with bouts of strong directional selection [14].

Hydrogen sulphide (H_2_S) is a widespread natural toxicant that can also originate from anthropogenic pollution, e.g., from pulp mills [15]. Its patchy occurrence often creates environmental gradients in aquatic ecosystems (both horizontally and vertically), e.g., at deep-sea hydrothermal vents, in coastal salt marshes, or in mudflats [16], [17]. H_2_S-toxicity results from its interference with mitochondrial respiration and blood oxygen transport, thereby inhibiting aerobic respiration, and this effect is aggravated by the fact that H_2_S leads to extreme hypoxia in the water [16], [17]. Accordingly, pulses of toxic H_2_S discharge have been reported to be the source of mass mortalities in aquatic faunas, especially fishes [17].

These adverse effects notwithstanding, a number of livebearing fishes (family Poeciliidae) have been documented to thrive in sulphide-rich habitats (reviewed in [18]). Patterns of trait divergence in these extremophile poeciliids and reproductive isolation towards neighbouring populations inhabiting non-toxic waters are congruent with ecological speciation as a response to adaptation to stressful environmental conditions and have been extensively studied in one particular system in southern Mexico. In this system, *Poecilia mexicana* inhabit various toxic and non-toxic surface habitats, but are also found in a subterranean ecosystem, the H_2_S-rich Cueva del Azufre [19], in which they are under strong selection from two extreme environmental forces: H_2_S-toxicity and permanent darkness. As a result, *P. mexicana* display considerable genotypic and phenotypic differentiation between adjacent extreme and benign habitat types [20]–[25]. Recent studies have demonstrated convergent patterns of morphological [26] and life-history diversification [27] (but see Riesch et al. submitted for non-convergent patterns), and similar mechanisms of selection against migrants from non-sulfidic habitats [28], [29] in *P. mexicana* from the Cueva del Azufre system and livebearing fishes inhabiting the Baños del Azufre, another sulfidic surface system in Tabasco, southern Mexico. These sulfidic springs are inhabited by two sulphur-endemics, the livebearing fishes *Gambusia eurystoma* and *Poecilia sulphuraria* [30]. The sulphur molly, *P. sulphuraria*, is of particular interest in this context, because phylogenetic analyses suggest that it is the sister-taxon to *P. mexicana* [26], [31] and therefore represents a possible ‘endpoint’ of ecological speciation in H_2_S-toxic surface habitats.

Several targets of divergent selection in extremophile poeciliids have recently been identified. On the life-history level, females from sulphidic, cave, or sulphur-cave habitats produce larger, but fewer offspring [23], [27], [32]–translating into increased individual offspring survivability [33]–and population differences in those traits have been demonstrated to have a heritable basis [24], [32]. Furthermore, the combined selection of H_2_S and darkness on offspring size in the Cueva del Azufre has resulted in *P. mexicana* from that cave having the largest offspring [23], [27]. Morphologically, extremophile livebearers exhibit convergent evolutionary trends primarily by developing larger heads and thus, larger branchial baskets than fish from non-toxic habitats [22], [26]. According to the species description by Alvarez [34], a species-specific trait of sulphur mollies are the dermal lip protuberances not found in any other poeciliid (Figure S1; please refer to the supporting figure legend). These protuberances are thought to enhance the performance of aquatic surface respiration (ASR; [30], [35]), a behavioural adaptation to meet oxygen demands in hypoxic habitats (e.g., [36], [37]). Cave mollies from the Cueva del Azufre are further characterized by smaller, yet fully functional eyes [22], [38]–[40] and reduced melanin pigmentation [38] (BL Joachim, I Schlupp, R Riesch, unpublished data).

### 1.2. Developmental patterns of divergent traits

Knowledge of development is paramount for understanding the mechanisms of evolutionary change, because changes in development can result in novel phenotypes [41], [42]. More specifically, changes in the timing of various developmental processes (i.e., heterochrony) may explain many of the evolutionary changes observed among closely-related taxa [43], [44]. Unfortunately, due to the inherent problem of acquiring embryonic and larval stages, developmental studies are rare in cave biology and research programmes focusing on evolutionary change in other extreme environments. One notable exception is the Mexican tetra, *Astyanax mexicanus*, which is easy to maintain in the laboratory, reproduces frequently, and therefore, is open to embryological/developmental investigations at the cellular and molecular levels [41], [42]. Albeit a single species, *A. mexicanus* has a surface-dwelling form that is widespread in the Atlantic slope of Mexico [45] and at least 30 known cave-dwelling forms that differ markedly from their surface-dwelling ancestors [41], [42], [46]. Typical for a variety of cave organisms, many cave-dwelling populations of *A. mexicanus* (often also referred to as *A. fasciatus*) have reduced eye size, while others have completely lost their eyes, and a similar pattern can be found for pigmentation [46]–[49]. In contrast to most other cave organisms, the developmental mechanisms underlying these changes in *A. mexicanus* are quite well understood (reviewed in [41], [42]).

A major obstacle for these types of developmental studies in extremophile poeciliids has been that they are livebearing, rendering the study of early developmental events difficult. Hence, even though the cave molly has been a model system for evolution of cave organisms for almost four decades now (reviewed in [38], [50]), and *P. sulphuraria* has recently become a model for ecological speciation processes as well (e.g., [18], [26], [27]), the question of how they achieve their divergent phenotypes during development has so far not been investigated. Convergent phenotypes (e.g., larger heads in extremophile *Poecilia*) are often, but not always, the result of convergent developmental modes [51]. Similar ecological conditions tend to produce convergent developmental modes, but few cases of convergence in development have been studied in detail. Wray [51] suggested that convergence in development is probably inversely related to phylogenetic distance with more closely related taxa also being more likely to exhibit convergent developmental modes (but see [52]). Our study system of *Poecilia* sister taxa that are characterized by several convergent phenotypic traits provides us with the perfect opportunity to test this assumption.

Here, we make use of the extensive data available to us from our own life-history analyses that were conducted on wild-caught fish for previous studies [23], [24], [27]. As part of the traditional life-history sampling protocol (see methods in [23], [24], [27], [53]), developing offspring were removed during the dissection of pregnant, formalin-fixed females and their stage of development determined according to Haynes [54] and Reznick et al. [55] (Table 1), and digital photographs were then taken of representative embryos for each stage of development. This provided us with excellent snap-shots of different stages of embryo development in all three mollies, and allowed us to track some of the most prominent divergent traits through development. In the present study, we examined embryos of surface-dwelling *P. mexicana* from benign surface habitats (henceforth called “surface mollies”), cave-dwelling *P. mexicana* from the toxic Cueva del Azufre (“cave mollies”), and the sulphur-endemic *P. sulphuraria* from a toxic spring (“sulphur mollies”) to examine at which points during development the local adaptations in life history and morphology would first be detectable and to what extent convergent phenotypes are the product of convergent developmental modes. Our study will provide an important starting point for future investigations into the molecular mechanisms of development, an approach that will also greatly benefit from the recently established protocol for *in vitro* culture of guppies, *Poecilia reticulata* [56]. Furthermore, results from transcriptomics studies will likely soon be available for extremophile *Poecilia* spp., so our present study may provide the basis for future studies linking gene expression patterns to developmental patterns. In this paper, we investigated the following four specific questions concerning the development of some key divergent traits in extremophile *Poecilia*:

1. When and how do cave and sulphur mollies increase offspring size at birth? Extremophile poeciliids could simply produce larger oocytes (i.e., provide more yolk to individual oocytes prior to fertilization), and embryos might then take roughly the same developmental route as smaller surface molly embryos. Another possibility is that extremophile poeciliids rely more on post-fertilization maternal nutrient transfer to embryos, in which case embryo size could either decrease to a lesser extent or even slowly increase during the course of development. Our first question, therefore, not only concerns embryo development *per se*, but also concerns the field of maternal provisioning, namely resource allocation pre- (vitellogenesis: [57]) and post-fertilization (matrotrophy: [58]–[62]).
2. Do cave mollies store fewer carotenoids in their ova? Cave organisms are usually characterized by reduced levels of carotenoids and carotenoid-derived compounds compared to surface-dwelling organisms, due to the scarcity of carotenoid-producing plants and microorganisms in subterranean habitats [63], [64]. However, a certain fraction of cave-dwelling organisms of various species are characterized by a distinct yellow body colouration that is thought to be the result of accumulated carotenoids [65]–[66] and cave mollies are no exception ([38]). In a field sample from January 2009, for example, approximately 26.6% (51 out of 192) of cave mollies from chamber V of the Cueva del Azufre were of the yellow colour variant (R Riesch, unpublished data). Since fish oocytes/ova obtain their characteristic golden to orange colouration from the presence of carotenoids [67], [68], measuring their yellowness should provide a reasonable proxy for the amount of carotenoids stored within the oocytes/ova.
3. How do extremophile mollies developmentally obtain their larger heads? This appears pretty straightforward and we therefore hypothesized that, compared to surface mollies, cave and sulphur mollies would boost head growth relative to the growth of other body parts early during development.
4. Finally, we asked how embryonic development leads to reduced eye size in cave mollies. Based on extensive work in *Astyanax* [41], [42] and some other cave fishes like the catfish *Rhamdia laticauda* [69], we predicted that the eye primordium in cave mollies should be slightly smaller compared to surface and sulphur mollies, and that eye growth in cave mollies should be arrested at a certain point during development [48].

**Table 1:** Embryo development in poeciliid fishes. According to Reznick [103] and compared to the generalized description provided by Haynes [54] (right).

Stage	Description	Stages according to [54]
0	mature (fully yolked) but unfertilised oocytes, even lipid distribution	stage 3
2	blastodisc embryos	stage 4
5	neurula stage: embryonic shield/primitive streak embryos	stage 5
10	optic cup embryos: head and optic cups are visible	stage 6
15	optic cups become pigmented (grey-eyed)	-
20	eyes appear fully pigmented (black) and caudal fin bud visible	stage 7
25	body pigmentation appears and pectoral fin buds	stage 8
30	caudal fin rays clearly visible, dorsal and anal fin buds	-
35	pectoral fin rays clearly visible	stage 9
40	dorsal and anal fin rays clearly visible	-
45	operculae conspicuous, scales visible, overall oval shape	stage 10
50	mature embryos: pericardial cavity almost or completely closed	stage 11

## Materials and Methods

### 2.1. Study populations and sampling protocols

Pregnant *P. mexicana* females were collected in June 2007 and January 2008 in two separate benign surface habitats (surface mollies; Río Amatan and Arroyo Bonita) and in the toxic Cueva del Azufre (cave mollies) –two sets of populations that are known to be reproductively isolated from one another [21], [22]. For more detail on these sample sites, please refer to Tobler et al. [22] and Riesch et al. [23]. Pregnant *P. sulphuraria* females were collected in August 2008 and January 2009 at the toxic Baños del Azufre (sulphur mollies; for more details, see [27]).

Following well-established life-history protocols [23], [27], [53], we removed all developing embryos from formaldehyde-preserved, pregnant females. Embryos were counted, their stage of development determined [53], [54], and then dried for 10 days at 40°C and reweighed. To assess embryo condition, embryos were rinsed six times for at least 6 h in petroleum ether to extract soluble nonstructural fats [70], and were then redried and reweighed. This protocol provided us with the following embryonic life history variables: embryonic dry weight [mg], embryonic lean weight [mg], and embryo fat content [%].

Prior to dissections, adult mollies were photographed with an InSight Spot 2 digital camera (Diagnostic Instruments) mounted on an Olympus SX 7 stereomicroscope and pictures were stored in TIFF file format. Immediately following surgical removal from preserved females, representative embryos for each molly and stage were also photographed and stored in TIFF file format.

### 2.2. Quantification of ovum yellowness

Embryo photographs were analyzed using Adobe Photoshop CS5 Extended 12.1 (Adobe Systems Inc., San Jose, CA, USA). TIFF images were opened in 16-bit mode and an L*a*b* colour space was employed [Commission International de l'Eclairage (CIE)]. In this colour space, b* values represent the ‘yellowness’ (balance between blue and yellow) of a photographic object (e.g., [71], [72]).

### 2.3. Quantifying maternal provisioning and the matrotrophy index

One potential pathway for extremophile *Poecilia* to achieve larger offspring size at birth would be to provide more post-fertilization maternal provisioning. To evaluate the mode of maternal provisioning, the matrotrophy index (MI) was calculated (utilizing the slopes and regression coefficients from the regression analysis described below). The MI equals the estimated dry mass of the embryo at birth divided by the estimated dry mass of the ovum at fertilization (e.g., [24], [55]). If the eggs were fully provisioned by yolk prior to fertilization (lecithotrophy), then we would expect the embryos to lose 35–40% of their dry mass during development (MI between 0.60 and 0.65; [58], [73]). On the other hand, in the case of continuous maternal provisioning even after fertilization (matrotrophy), one would expect the embryos to lose less weight (MI between 0.65 and 1.00) or to even gain weight during development (MI>1.00; e.g., [55]). Thus, maternal provisioning was evaluated by analyzing the relationship between log-transformed embryonic dry mass and stage of development by means of linear regression analysis [55]. For this analysis, data for one of the two species examined, namely *P. mexicana* (surface and cave mollies), were largely reanalyzed from two previous studies [23], [24], but new data were also included.

### 2.4. Measuring ovum diameter and collection of morphological data

Using Spot Imaging Software, version 4.5 (Diagnostic Instruments), photos were size calibrated for the specific microscope magnification used to take each picture. Then, using the ‘measurements’ function integrated into Spot Imaging Software, we measured the following traits: ovum diameter [mm] (maximum diameter of ovum and/or embryo), eye diameter [mm], head length [mm] (from tip of embryo mouth to end of head capsule), and body length [mm] (from tip of embryo mouth to caudal peduncle). We then used these traits to calculate two indices, “relative eye size (eye diameter divided by body length)” and “relative head size (head length divided by body length)”. These two indices were also calculated for measurements taken from ten randomly selected photographs of adult mollies.

### 2.5. Statistical analyses

Previous studies have demonstrated that the population means for the traits reported here are similar between surface molly populations from southern Mexico (i.e., Río Amatan and Arroyo Bonita) [22]–[25] and thus both were pooled to increase sample size and statistical power.

For the analyses on information extracted directly from the embryonic pictures, for each developmental stage we used offspring from 2.33±1.07 (mean±SD) females in surface mollies, 1.67±0.65 females in cave mollies, and 1.75±0.62 females in sulphur mollies. In other words, some data points for any given stage are from embryos of the same clutch, while other data points are from embryos of different clutches. The analyses on embryo weight and fat, however, utilized data from a much larger data set. Here, each data point is fully independent and represents the mean for all embryos of a single clutch.

For embryos of all three types of mollies, we conducted six separate ANCOVAs. For each model “stage of development” served as the covariate and “ecotype (surface molly vs. cave molly vs. sulphur molly)” the independent variable. To test for heterogeneity of slopes, the interaction of “ecotype by stage of development” was also included. The dependent variables in the different ANCOVA models were “yellowness of the yolk”, “embryo dry weight”, “ovum diameter”, “embryo fat content”, “relative eye size”, and “relative head size”, respectively.

For adult mollies we conducted two separate one-way-ANOVAs to investigate differences between ecotypes. The first tested for differences in “relative eye size” and the second for differences in “relative head size”. In case of significant differences among groups, we then conducted Fisher's LSD post hoc tests.

All statistical analyses were conducted with PASW Statistics 18.0.3 (IBM SPSS).

## Results

### 3.1. General embryo development

**Figure 1:**
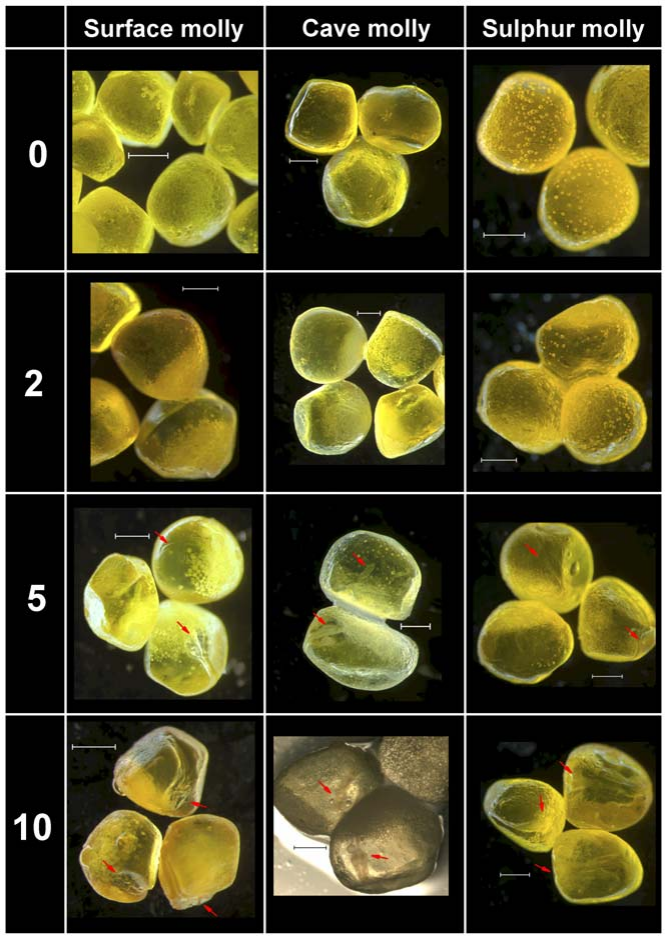
Comparative embryo development of two ecotypes of *P. mexicana* and of *P. sulphuraria*. Stages range from the unfertilised, but ripe oocyte (Stage 0), to the optic cup embryo (Stage 10). See Table 1 for details. Scale bar  =  1 mm. Embryos of stage 2 are lateral view. Arrows at stage 5 indicate location of the primitive streak embryo; arrows at stage 10 indicate location of head and optic cups.

**Figure 2:**
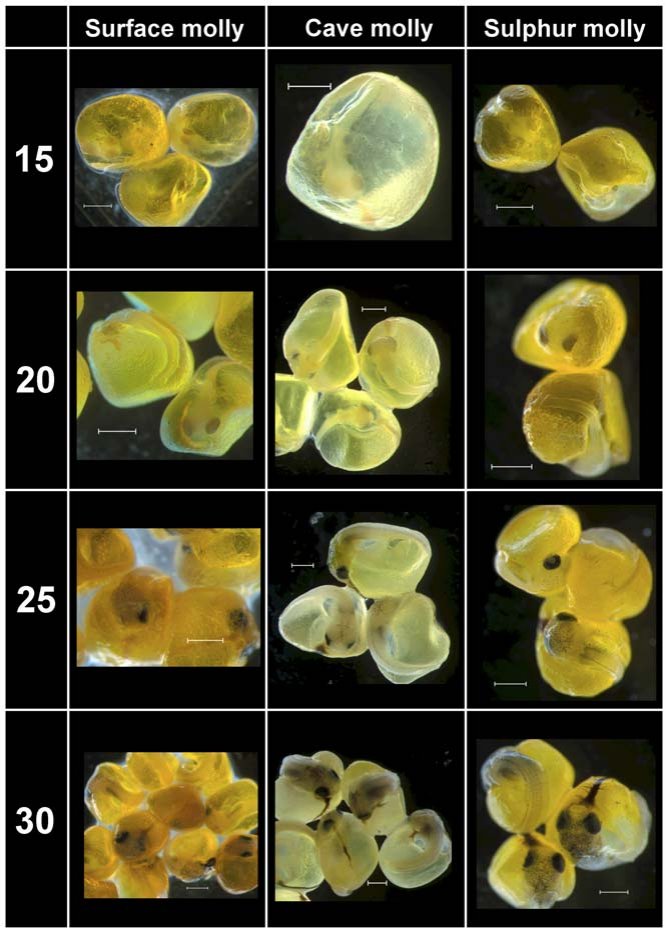
Comparative embryo development of two ecotypes of *P. mexicana* and of *P. sulphuraria*. Stages range from Stage 15 to Stage 30. See Table 1 for details. Scale bar  =  1 mm.

**Figure 3:**
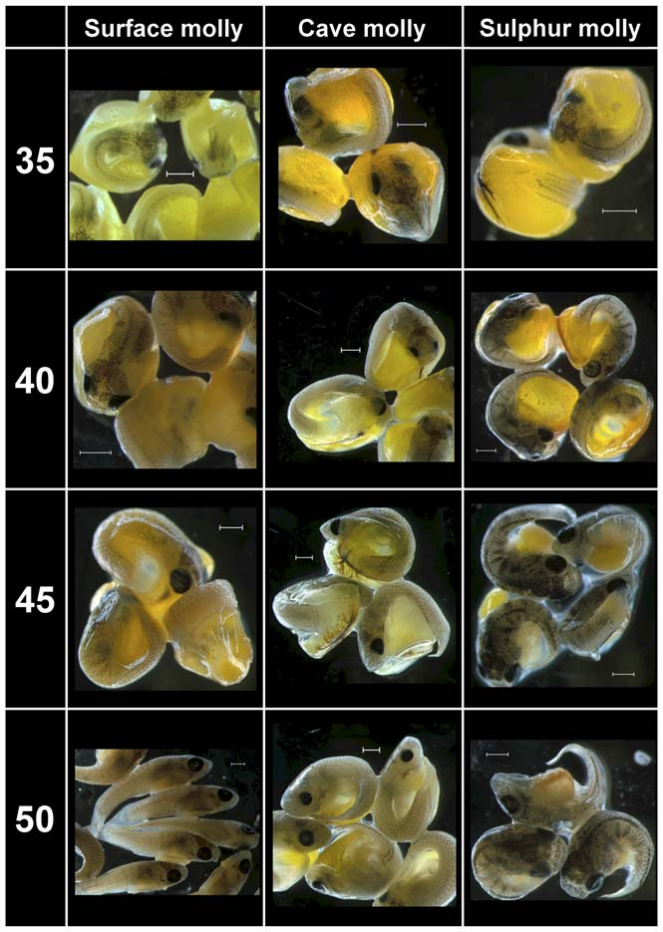
Comparative embryo development of two ecotypes of *P. mexicana* and of *P. sulphuraria*. Stages range from Stage 35 to the ready-to-be-born embryo (Stage 50). See Table 1 for details. Scale bar  =  1 mm.

In agreement with the standardized protocol (Table 1), development of eye pigmentation progressed in a similar fashion starting with the unpigmented optic cups in stage 10 embryos all the way to fully pigmented eyes in stage 20 embryos (Figures 1 and 2). Between stages 20 and 25 the first melanophores appeared in all embryos, including cave mollies (Figure 2). Body pigmentation first appeared above the brain in stage 25 and then appeared along the horizontal dorsal midline (Stages 25 and 30; Figure 2). In general, melanophore pigmentation in cave molly embryos followed similar developmental trajectories as in surface or sulphur molly embryos; however, at stages 40–50 cave molly embryos appeared slightly paler, and individual pigment cells seemed to be smaller than in both other mollies (Figure 3), suggesting a decrease in pigmentation late in development for cave mollies.

While 31 out of 45 (69%) *P. sulphuraria* adults had characteristic lower lip protuberances (Figure S1; please refer to the Supporting Figure Legend), we found no evidence for lip protuberances even in late-stage *P. sulphuraria* embryos (Figure 3).

### 3.2. Yellowness of the yolk

**Figure 4:**
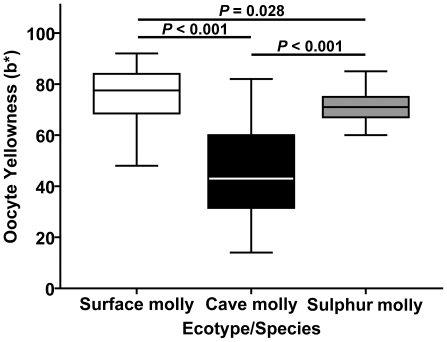
Ovum yellowness as a proxy for carotenoid content. Measured as the b* component of the CIE l*a*b* colour mode in Adobe^©^ Photoshop^©^ CS5. Box plots (median and IQR). Yellowness was measured for all stages combined since a preliminary ANCOVA revealed that developmental stage was not correlated with ovum yellowness. Surface mollies, *N* = 72; cave mollies, *N* = 47; sulphur mollies, *N* = 50. ANCOVA details are presented in the results section; *p*-values refer to Fisher's LSD post hoc tests.

Neither the covariate “developmental stage” (*F*_1,163_ = 0.00, *P* = 0.99) nor the interaction of “ecotype by developmental stage” (*F*_2,163_ = 1.11, *P* = 0.33) had an effect on the yellowness of *Poecilia* ova, but the model clearly revealed a significant difference between ecotypes (*F*_2,163_ = 39.88, *P*<0.001). Since neither the covariate nor the interaction terms were significant, we conducted post hoc tests from a simplified model that only included the factor “ecotype”. This analysis revealed that all three mollies differed significantly from one another (Fisher's LSD, surface vs. sulphur: *P* = 0.028; all other comparisons: *P*<0.001; Figure 4).

### 3.3. Embryo development: how to increase offspring size

#### 3.3.1. Embryo mass and matrotrophy index (MI)

**Figure 5:**
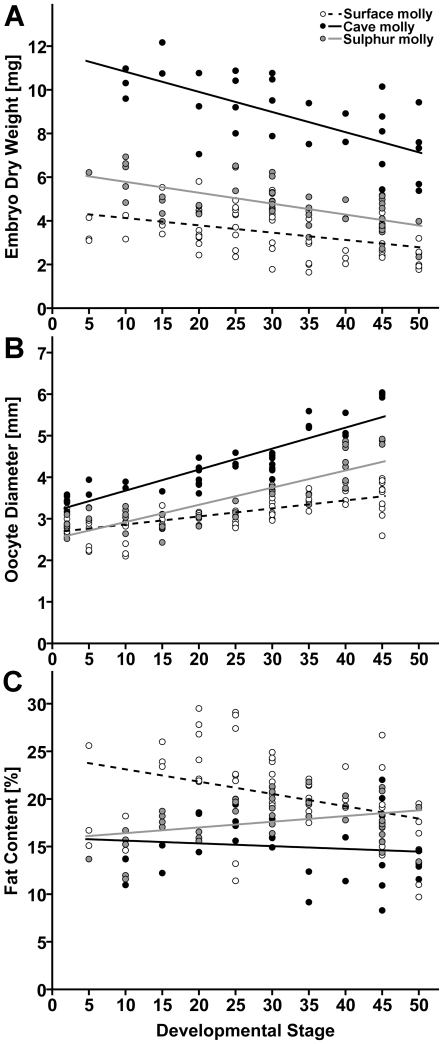
How to increase offspring size during development. Scatter plots depicting (**A**) embryo dry weight, (**B**) ovum diameter, and (**C**) embryo fat content in the course of development in molly embryos from one benign and two extreme habitats. Test statistics are presented in the results section.

Embryos lost weight during the course of gestation in all three mollies (“developmental stage”: *F*_1,132_ = 44.95, *P*<0.001); we did not find a significant interaction of “ecotype by developmental stage” though (*F*_2,132_ = 0.00, *P* = 0.99), suggesting no heterogeneity of slopes (surface mollies, *N* = 63; cave mollies, *N* = 31; sulphur mollies, *N* = 44; Figure 5A). However, the three mollies differed drastically from each other in embryo mass (“ecotype”: *F*_2,132_ = 24.38, *P*<0.001), with cave molly embryos being by far the heaviest, sulphur molly embryos being intermediate in dry mass, and surface molly embryos being the lightest (Figure 5A). This also resulted in different estimated MI values for all three mollies despite similar slopes (surface molly  =  0.62; cave molly  =  0.77; sulphur molly  =  0.69; Figure 5A). In other words: sulphur and especially cave mollies initiated development with heavier eggs, but all employed a similar, mainly lecithotrophic provisioning strategy.

#### 3.3.2. Ovum/embryo diameter

“Ecotype” (*F*_2,141_ = 15.48, *P*<0.001), “developmental stage” (*F*_1,141_ = 236.35, *P*<0.001), and the interaction of “ecotype by developmental stage” (*F*_2,141_ = 9.54, *P*<0.001) all had significant influence on ovum/embryo diameter (surface mollies, *N* = 69; cave mollies, *N* = 37; sulphur mollies, *N* = 41; Figure 5B). While the diameter increased in all three mollies, it did so to a much stronger extent in the two extremophile mollies. Furthermore, surface mollies and sulphur mollies appear to have approximately similar sized ripe ova at the point of fertilization, while cave mollies initiated development with larger unfertilized ova (Figure 5B). (Note the apparent discrepancy to the pattern seen in ovum mass at fertilization, which was higher in sulphur than surface mollies, see above).

The ovum diameter at stage 2 was significantly different between ecotypes (surface mollies, *N* = 9; cave mollies, *N* = 5; sulphur mollies, *N* = 4; ANOVA: *F*_2,15_ = 6.46, *P* = 0.009) and post-hoc tests revealed that this was due to significant differences between cave mollies and both other ecotypes (Fisher's LSD, cave vs. surface: *P* = 0.015; cave vs. sulphur: *P* = 0.004; surface vs. sulphur: *P* = 0.224).

#### 3.3.3. Embryo fat content

Embryos of *P. mexicana* lost fat during the course of development, albeit at different rates, while sulphur molly embryos actually gained body fat during development. Accordingly, we found significant effects of “ecotype” (surface mollies, *N* = 63; cave mollies, *N* = 31; sulphur mollies, *N* = 44; *F*_2,132_ = 13.11, *P*<0.001) and the interaction of “ecotype by developmental stage” (*F*_2,132_ = 5.77, *P* = 0.004) on fat content; however, the effect of “developmental stage” was not significant (*F*_1,132_ = 1.75, *P* = 0.19; Figure 5C). Surface molly embryos began development with the highest percentage of body fat, but at around stage 40 fat content of surface and sulphur mollies converged. Cave mollies, on the other hand, had the lowest fat content of all three mollies throughout development (Figure 5C).

### 3.4. Embryo development: how to achieve divergent morphologies

#### 3.4.1. Relative head size

**Figure 6:**
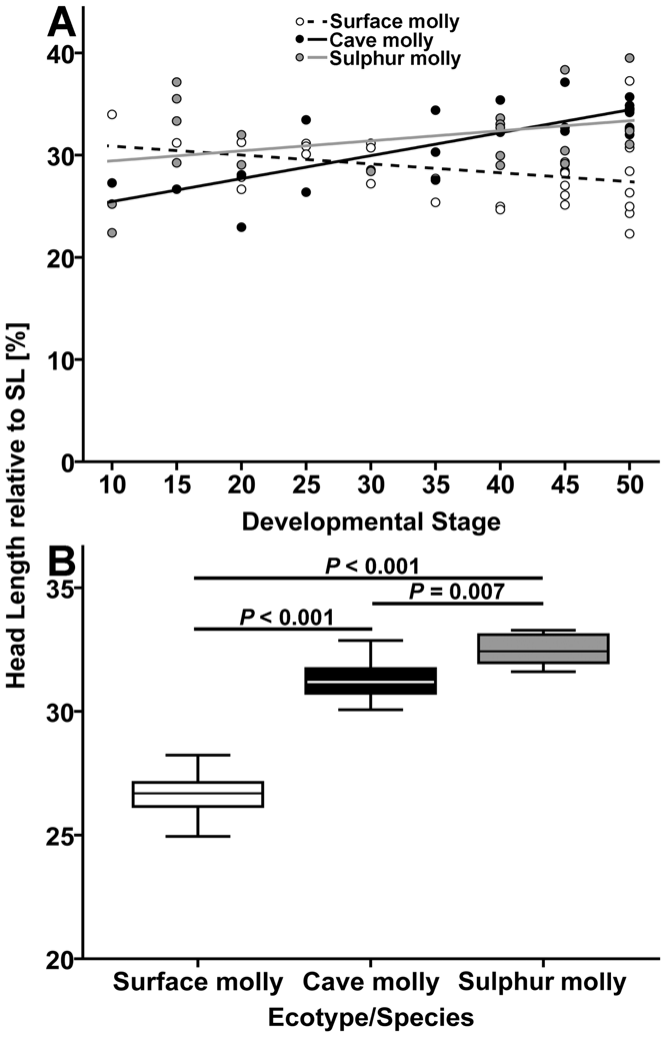
Relative head length in three types of mollies. (**A**) Scatter plots showing the ‘head length to SL’ ratio vs. stage of development in molly embryos from one benign and two extreme habitats. Stages 0, 2 and 5 are not depicted, because they lack a differentiated head. (**B**) Box plots (median and IQR) for the head length to SL ratio in adult fish (*N* = 10 per population). *P*-values refer to Fisher's LSD post hoc tests. Test statistics for both graphs are presented in the results section.

In the ANCOVA (surface mollies, *N* = 31; cave mollies, *N* = 23; sulphur mollies, *N* = 20) on head length to SL ratio across embryo developmental stages, both the factor “ecotype” (*F*_2,68_ = 4.79, *P* = 0.011) and the covariate “developmental stage” (*F*_1,68_ = 7.66, *P* = 0.007) had a significant influence, but the interaction of “ecotype by developmental stage” was also significant (*F*_2,68_ = 10.19, *P*<0.001; Figure 6A). Inspection of the raw data indicates that relative head length decreases in surface mollies, but increases in cave and sulphur mollies (Figure 6A).

The ratio of ‘head length to SL’ was significantly different between adults of the three ecotypes (*N* = 10 for all mollies; *F*_2,27_ = 72.50, *P*<0.001) and post-hoc tests revealed that this was due to significant differences between all three mollies (Fisher's LSD, cave vs. surface and surface vs. sulphur: *P*<0.001; cave vs. sulphur: *P* = 0.007; Figure 6B).

#### 3.4.2. Relative eye size

**Figure 7:**
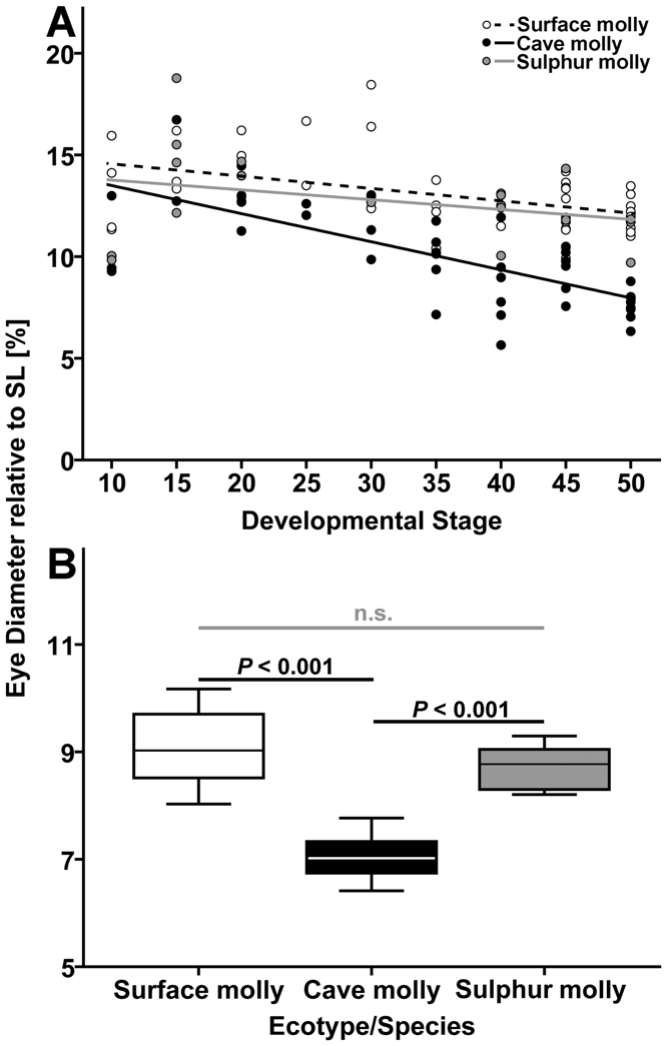
Relative eye size in three types of mollies. (**A**) Scatter plots of eye diameter to SL ratio vs. stage of development in molly embryos from one benign and two extreme habitats. Stages 0, 2 and 5 are not depicted, because they lack a differentiated head. (**B**) Box plots (median and IQR) for the ‘eye diameter to SL’ ratio in adult fish (*N* = 10 per population). *P*-values refer to Fisher's LSD post hoc tests. Test statistics for both graphs are presented in the results section.

ANCOVA (surface mollies, *N* = 43; cave mollies, *N* = 45; sulphur mollies, *N* = 21) revealed significant effects of the covariate “developmental stage” (*F*_1,103_ = 39.16, *P*<0.001) and “ecotype by developmental stage” (*F*_2,103_ = 4.94, *P* = 0.009) on the ‘eye diameter to SL’ ratio across embryo development; however, the effect of “ecotype” was not significant (*F*_2,103_ = 0.28, *P* = 0.75; Figure 7A). Clearly, relative eye size decreases with increasing developmental stage in all populations, but closer inspection also suggests that the interaction effect is due to the stronger decrease in relative eye size in cave mollies compared to the other two populations (Figure 7A). Comparison of the absolute eye diameter between mid-stage (stage 35) and late-stage (stage 50) embryos revealed that this strong decrease in relative eye size was due to an arrest of eye growth in cave mollies, in which the median eye diameter at stage 35 measured 0.81 mm (IQR = 0.19 mm) versus 0.72 mm (IQR = 0.16 mm) at stage 50 (Mann-Whitney *U*-test: *U*_12_ = 14.50, *P* = 0.37). By contrast, eyes still grew larger in surface mollies (*U*_13_ = 40.00, *P* = 0.002); the median eye diameter at stage 35 was 0.74 mm (IQR = 0.11 mm), and 1.09 mm (IQR = 0.18 mm) at stage 50. Due to limited sample size, this comparison was not possible for sulphur mollies.

The ratio of ‘eye diameter to SL’ was significantly different between adults of the three different mollies (*N* = 10 for all mollies; *F*_2,27_ = 31.00, *P*<0.001) and post-hoc tests revealed that this was due to cave mollies having smaller eyes than either surface mollies (Fisher's LSD: *P*<0.001) or sulphur mollies (*P*<0.001; Figure 7B).

## Discussion

### General embryo development

Pigmentation patterns are thought to play a major role in speciation events because they are important visual cues in species recognition, mate choice, and predator avoidance [74]. Cave organisms, usually living in constant darkness, are often characterized by the absence or reduction in melanin body pigmentation [75]. It has long been established that cave mollies, when caught in the Cueva del Azufre, are devoid of most visible pigmentation patterns otherwise characteristic for *P. mexicana* ([19]; reviewed in [38], [50]). Nonetheless, once exposed to light in the laboratory environment, cave mollies partly regain the species-specific colour patterns over a period of several weeks, and a recent analysis revealed that, while their numbers are reduced compared to surface mollies, cave mollies do have melanophores (BL Joachim, I Schlupp, R Riesch, unpublished data). Our results are congruent with this pattern, because cave molly embryos clearly develop melanophores just like other surface-dwelling poeciliids at around stage 25, but in late-stage cave molly embryos overall strength of pigmentation seems to be reduced compared to surface and sulphur mollies. While the genetic basis and fine-scale developmental pathways of pigmentation loss in *Astyanax* cavefish are well understood (e.g., [76]; reviewed in [41], [42]), future studies will have to establish expression patterns of developmental genes in cave mollies.

Modified lips with dermal protuberances are deemed a species-specific, apomorphic character of sulphur mollies [34], [45]. Clearly these protuberances are traits that are unique to sulphur mollies despite the fact that other poeciliids (including the cave molly) also inhabit similarly toxic and hypoxic habitats; however, as we demonstrated here, only a fraction of adult fish actually exhibit them at any given time. Furthermore, our experience from the field suggests that these are highly plastic traits, because adult fish with prominent lip protuberances caught at the Baños del Azufre regularly loose them within hours to days of being introduced to non-sulphidic laboratory conditions (M Plath, R Riesch, I Schlupp, personal observation). Thus, they are similar to other fishes from (temporarily) hypoxic habitats, in which lip protuberances are also reversible and usually only a certain percentage of fish within a population display the trait at any given time (e.g., [35]). This high degree of phenotypic plasticity also best explains why we found no evidence for lip protuberances in any of the sulphur molly embryos. We propose that this trait may be developed ‘at will’ if the environmental conditions (i.e., sulphide-toxicity and low oxygen levels) require it. Some sulphur mollies are certainly born into less sulfidic microhabitats at the Baños del Azufre, and these lip protuberances are likely to lead to hydrodynamic disadvantages as a result of increased drag, so not expressing the trait already within the mother's body cavity may actually be beneficial.

Carotenoids serve several important functions in animals, ranging from being Vitamin A precursors and providing the basis for body coloration patterns to providing the precursors for the visual pigment retinal and acting as antioxidants and immune-system enhancers ([77]–[80]; but see [76]). Animals cannot synthesize carotenoids *de novo* and consequently, have to acquire them from their diet [67]. In fish, eggs usually obtain their characteristic golden to orange coloration from the presence of carotenoids stored in the oocytes [67], which has been experimentally confirmed by Hubbs and Strawn in their study on darters [65]. The presence of low levels of carotenoids in cave organisms has long been established [63], [64] and it is therefore not surprising that cave mollies are able to produce pigmented eyes and (reduced) body pigmentation. Our results demonstrate that although cave molly oocytes are still yellow, the intensity of the yellow colour component is significantly reduced relative to surface and sulphur mollies, suggesting reduced carotenoid content in the yolk of cave molly oocytes. Compared to regular surface habitats that are filled with carotenoid-producing plants, algae and bacteria, carotenoids will be relatively scarce in the natural cave environment. So, where do the carotenoids come from in the Cueva del Azufre? Several mutually non-exclusive scenarios are possible. First, in several parts of the cave, skylights introduce dim light into several cave chambers [19], so it may be possible that carotenoid synthesizing algae and bacteria grow in certain parts of the Cueva del Azufre, in particular since even chemoautotroph sulphur-oxidizing bacteria have been described to produce carotenoids [81]. Other significant sources of carotenoids in caves stem from allochthonous input in the form of fresh plant material that falls through skylights, and detritus carried in by underground rivers [63]. Carotenoids are also found in chironomid eggs and larvae [82], which are numerous in the Cueva del Azufre and are known to be an important food source for cave mollies [83], even though stable isotope analysis suggests that cave mollies assimilate material obtained from chironomid eggs and larvae only to minor proportions into body tissue [84].

Furthermore, ovum yellowness also differed significantly between surface and sulphur mollies, with sulphur molly ova having slightly reduced yellowness. This is most likely due to the fact that poeciliids in sulphidic surface habitats primarily feed on detritus, arthropods, and bacteria, while surface mollies primarily feed on detritus and algae [83], [84]. Even though chemoautotrophic sulphur-oxidizing bacteria have been described to produce carotenoids [81], it is unlikely that they are produced in comparable amounts as in nontoxic surface habitats, where carotenoids are synthesized by algae and other photoautotrophic primary producers.

However, we cannot exclude the possibility that some of the variation in ova coloration is due to additional differences in lipid and protein content between ecotypes and species [85]. Clearly, this would also be influenced by the available resources in each habitat and could slightly confound our results. Future experiments investigating the actual carotenoid, lipid and protein content of poeciliid ova are needed to clearly resolve this.

Finally, in teleost fishes offspring viability correlates positively with oocyte/ovum carotenoid content, with young born from pale ova of low carotenoid content experiencing increased levels of juvenile mortality [86]–[88]. Our results on ovum yellowness suggest that cave mollies have locally adapted to the reduced carotenoid availability and appear to have evolved a mechanism to offset the cost of reduced carotenoid levels on offspring viability, because cave molly offspring actually have a higher individual fitness compared to surface molly offspring [33]. Hence, the decreased availability of carotenoids likely results in strongly decreased fitness in immigrant fish not showing specific adaptations to these conditions and thus helps maintain the strong reproductive isolation detected in this system [20]–[22], [28], [89].

### Embryo development: how to increase offspring size

All three mollies were characterized by lecithotrophy according to the MI analysis. However, a recent comparative analysis of maternal provisioning in surface and cave mollies, based on the detection of transferred radioactively labelled leucine from mothers to embryos, demonstrated that low levels of postfertilization provisioning are possible even if MI values point towards lecithotrophy [24]. It is therefore highly likely that also sulphur mollies are characterized by dual provisioning rather than pure lecithotrophy (see also [62]). Nonetheless, cave and sulphur mollies clearly show unique developmental patterns to achieve larger embryo size at birth. Cave mollies produced larger ova prior to fertilization and ovum diameter then steadily increased throughout development, mainly driven by the large embryos that, starting around stage 20, led to increasing ovum/embryo diameter. Sulphur mollies, on the other hand, produced ova that at fertilization were not larger than those of surface mollies and only started to diverge at later stages during development (again around stage 20). It is important to keep in mind though, that we only measured ovum diameter on the photos taken of developing embryos rather than on ova that had the developing embryos removed prior to the measurement being taken. This obviously confounded our measurements, because at later stages only small parts of the ovum were visible and the diameter was basically determined as the maximum diameter measurable on ovum or developing embryo and so relatively larger embryos will have confounded our measure of ovum diameter. Nonetheless, this pattern for sulphur mollies is highly surprising, because sulphur molly offspring are significantly heavier throughout development. Similarly surprising is the pattern observed for embryo fat content during development. It decreased in both ecotypes of *P. mexicana* (i.e., surface and cave mollies), albeit to different degrees, but actually increased during development in sulphur mollies. How can the surprising patterns discovered in sulphur mollies be explained? We can only speculate at this point and future research will have to test our hypotheses. Apparently, sulphur mollies are able to create ova with an increased density of nutrients, which would explain how ova could be heavier, yet at the same time be of the same diameter as surface molly ova. Furthermore, we hypothesize that sulphur molly embryos are actually converting some of the nutrients originally stored in the ova into soluble body fats during embryo development. This would explain why sulphur molly embryos are the only ones in which fat content actually increased during development even though overall embryo weight continuously decreased. This pattern is congruent with recent life-history analyses on adult sulphur mollies [27] (R Riesch, M Plath, I Schlupp, RB Langerhans, unpublished data) that revealed sulphur mollies to have surprisingly high levels of body fat that even exceed those of surface mollies from benign habitats. Clearly, at this point in time we can only speculate on the underlying mechanisms for this; however, one has to keep in mind that sulphur and cave mollies are the result of independent colonizations of sulphur habitats. Furthermore, the two habitats are quite different in their physical characteristics. The cave is not completely linear; in other words not all parts of all cave chambers are immediately downstream of the toxic springs, and the innermost cave chamber (XIII) is actually nontoxic [19]. So, cave mollies actually have a better opportunity to escape into microhabitats that are less toxic if the hydrogen sulphide discharge should temporarily increase. Sulphur mollies, in contrast, do not have this option to a similar extent. Almost their entire habitat is immediately downstream of the toxic springs, and if they evade toxicity too much they increase interspecific competition with another sulphur-endemic poeciliid (the widemouth gambusia, *Gambusia eurystoma*) that inhabits the more peripheral parts of the Baños del Azufre [27], [30]. Given these and (potentially) additional differences (i.e., due to genetic drift), we do not find it unlikely that sulphur mollies evolved a coping mechanism that is lacking in cave mollies. Thus, we hypothesize that at some point during the evolutionary history of the sulphur molly, divergent metabolic pathways must have evolved that enable sulphur mollies to more efficiently create body fat out of available resources (for a similar phenomenon in a variety of cave organisms–but not cave mollies–from resource-limited habitats see [90]). Potentially, these fat reserves could function as an ATP reservoir that can be tapped into during periods of high temporal hydrogen sulphide discharge, and thus, provides sulphur mollies with the energy required to immediately up-regulate physiological detoxification mechanisms.

We were able to demonstrate in previous studies that differences in offspring size are not simply a function of differences in female body size between species and ecotypes but rather represent true (evolved) differences [23], [27]. Furthermore, the patterns are actually opposite to what would be expected, as cave molly females are on average smaller than surface molly females [23], and sulphur molly females are smaller still [27]; nonetheless, both ecotypes/species have larger offspring than surface molly females.

Naturally, some of the differences detected here not only represent the action of evolved (genetic) differences but also environmental factors like resource availability. Since our embryo development data are derived from pregnant females collected in their natural habitat, they represent snap-shots of embryo development in the wild and are therefore also subject to phenotypic plasticity induced by environmental differences. For example, an important factor in shaping offspring phenotype is variation in parental nutrient provisioning, which, in turn, depends on environmental conditions [59], [91]. Still, given that population differences in the traits considered here have been shown to be largely heritable in adults [22], [24], [38], we argue that most of the differences observed here are unlikely to solely represent the effects of phenotypic plasticity.

### Embryo development: how to achieve divergent morphologies

Not only cave mollies [22], [26], but also various other cave organisms, like cave-adapted fishes of the North American family Amblyopsidae or the cave salamander *Proteus anguinus* show enlarged heads [92], [93]. In amblyopsids, in which both surface and several cave forms exist, it was suggested that heads become relatively larger with increasing cave adaptation, and this was interpreted as a neotenic trend that enables sensory adaptations to life in darkness. For example, an enlarged head surface area could facilitate more superficial neuromasts [93]. Our studies on *Poecilia* spp. do not support this view, as also surface fish from sulphidic surface habitats evolve larger heads [22], [26]. As cave ecosystems naturally do not show any photosynthetic oxygen production, hypoxia is common [94], [95]. We therefore argue that the necessity to improve oxygen uptake via enlarged gills has selected for enlarged branchial baskets and thus, larger heads not only in extremophile poeciliids, but several other cave organisms as well.

All sightless cave-dwelling vertebrates investigated to date exhibit initial eye development followed by secondary degradation during later developmental stages [41], [42], [68], [96], [97], because early steps in eye and pigment development are thought to be vital for other essential steps in development [41], [42]. In cave-dwelling *Astyanax* and *Rhamdia*, early eye development already starts with a smaller eye primordium, and culminates in subsequent eye growth arrest [41], [42], [68]. Even though cave molly adults have reduced eye size compared to surface and sulphur mollies, this reduction in eye size appears to be mainly the product of growth arrest during the later developmental stages (stage 35 – stage 50) rather than resulting from disproportionally smaller eye primordia at stage 10. Clearly, cave mollies are an evolutionarily young lineage compared to some eyeless *Astyanax* populations, so growth arrest might be the developmental trait that is easiest to manipulate in the early stages of the evolution of cave organisms; however, we cannot rule out that at a later point during cave evolution, cave mollies will also evolve means to reduce the size of the eye primordium.

### Conclusions

The present study demonstrates that most traits (convergent and unique) under strong selection in extreme habitats diverge early in development in extremophile *Poecilia*, indicating that both cave and sulphur mollies are in the advanced stages of ecological specialization. Thus, the present study is further evidence for ecological speciation in extremophile poeciliids.

Furthermore, certain phenotypic traits (i.e., larger heads) appear to be the result of largely convergent developmental modes among extremophile fish, while other convergent traits (i.e., larger offspring size at birth) seem to be the result of taxon-specific modifications in embryo development and nourishment. Numerous studies have investigated to what extent phenotypic divergence to shared selective regimes is convergent or species/population-specific (i.e., unique) among closely related organisms or even different sexes within a species (e.g., [98]–[101] (R Riesch, M Plath, I Schlupp, RB Langerhans, unpublished data). However, rarely are the developmental modes responsible for convergent adult phenotypes examined in a comparative approach (but see for example [51]). Our results clearly demonstrate that convergent shifts in phenotypic traits among closely related taxa are not always achieved by convergent processes during embryo development, but rather, that even sister taxa can evolve taxon-specific, alternative developmental modes to achieve replicated phenotypes [102]. Our study supports the view forwarded by Arendt & Reznick [52], who suggested that scientists should refrain from making the distinction between parallel and convergent evolution, because even closely related taxa can evolve replicated phenotypes via alternative developmental and genetic mechanisms, while distantly related taxa sometimes evolve replicated phenotypes via the same developmental and genetic mechanisms (reviewed in [52]; but see also [102]).

### Ethics Statement

The experiments comply with the current laws on animal experimentation of the United States of America and were approved by the Institutional Animal Care and Use Committee of the University of Oklahoma (AUS- IACUC approved protocols: R06–026 and R09–023).

## Supporting Information

Figure S1 **Lip protuberances of the sulphur molly (** ***P. sulphuraria*** **).** Female, dorsal view. Arrows indicate lip protuberances. Scale bar  =  1 mm.
(TIF)
