# Supplementary figures and images for: Shared and Unique Patterns of Embryo Development in Extremophile Poeciliids
Source: PLoS One. 2011 Nov 7;6(11):e27377. doi: 10.1371/journal.pone.0027377 (PMC3210165; doi:10.1371/journal.pone.0027377)

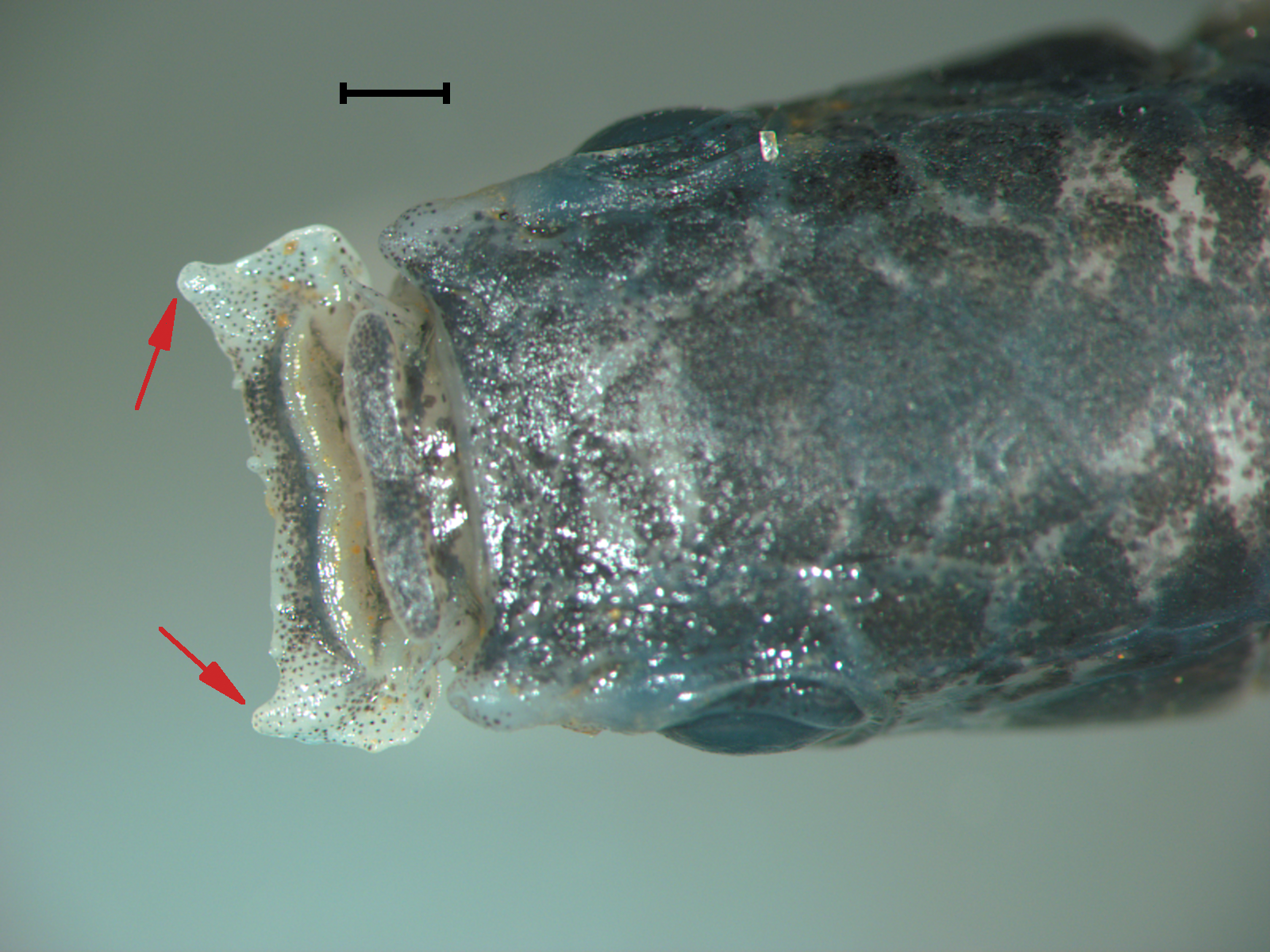

Supplement: Figure S1 — Lip protuberances of the sulphur molly ( P. sulphuraria ). Female, dorsal view. Arrows indicate lip protuberances. Scale bar = 1 mm. (TIF) [file pone.0027377.s001.tif]
